# Multi-Targeted Neutron Capture Therapy Combined with an 18 kDa Translocator Protein-Targeted Boron Compound Is an Effective Strategy in a Rat Brain Tumor Model

**DOI:** 10.3390/cancers15041034

**Published:** 2023-02-06

**Authors:** Hideki Kashiwagi, Yoshihide Hattori, Shinji Kawabata, Ryo Kayama, Kohei Yoshimura, Yusuke Fukuo, Takuya Kanemitsu, Hiroyuki Shiba, Ryo Hiramatsu, Toshihiro Takami, Takushi Takata, Hiroki Tanaka, Tsubasa Watanabe, Minoru Suzuki, Naonori Hu, Shin-Ichi Miyatake, Mitsunori Kirihata, Masahiko Wanibuchi

**Affiliations:** 1 Department of Neurosurgery, Osaka Medical and Pharmaceutical University, Osaka 569-8686, Japan; 2 Research Center of BNCT, Osaka Metropolitan University, Osaka 599-8531, Japan; 3 Institute for Integrated Radiation and Nuclear Science, Kyoto University, Osaka 590-0494, Japan; 4 Kansai BNCT Medical Center, Osaka Medical and Pharmaceutical University, Osaka 569-8686, Japan

**Keywords:** boron neutron capture therapy (BNCT), high-grade gliomas (HG), 18 kDa translocator protein (TSPO), convection-enhanced delivery (CED), DPA

## Abstract

**Simple Summary:**

In recent years, boron neutron capture therapy (BNCT) has attracted attention as a treatment for high-grade gliomas, and clinical trials using an accelerator-based neutron source have shown its effectiveness. However, high-grade gliomas are still diseases that cannot be cured, and drug discovery of novel boron compounds has been actively pursued. We investigated the feasibility of boron neutron capture therapy targeting the 18 kDa translocator protein (TSPO), which is expressed in glioblastoma and surrounding environmental cells. In combination with an existing drug, boronophenylalanine (BPA), we obtained an add-on effect to neutron capture therapy in an experimental F98 rat glioma bearing brain tumor model. The potential to provide multi-targeted neutron capture therapy by combining boron compounds with different biological targeting properties was demonstrated.

**Abstract:**

Background: Boron neutron capture therapy (BNCT) has been adapted to high-grade gliomas (HG); however, some gliomas are refractory to BNCT using boronophenylalanine (BPA). In this study, the feasibility of BNCT targeting the 18 kDa translocator protein (TSPO) expressed in glioblastoma and surrounding environmental cells was investigated. Methods: Three rat glioma cell lines, an F98 rat glioma bearing brain tumor model, DPA-BSTPG which is a boron-10 compound targeting TSPO, BPA, and sodium borocaptate (BSH) were used. TSPO expression was evaluated in the F98 rat glioma model. Boron uptake was assessed in three rat glioma cell lines and in the F98 rat glioma model. In vitro and in vivo neutron irradiation experiments were performed. Results: DPA-BSTPG was efficiently taken up in vitro. The brain tumor has 16-fold higher TSPO expressions than its brain tissue. The compound biological effectiveness value of DPA-BSTPG was 8.43 to F98 rat glioma cells. The boron concentration in the tumor using DPA-BSTPG convection-enhanced delivery (CED) administration was approximately twice as high as using BPA intravenous administration. BNCT using DPA-BSTPG has significant efficacy over the untreated group. BNCT using a combination of BPA and DPA-BSTPG gained significantly longer survival times than using BPA alone. Conclusion: DPA-BSTPG in combination with BPA may provide the multi-targeted neutron capture therapy against HG.

## 1. Introduction

Boron neutron capture therapy (BNCT) has attracted attention for therapy against high-grade gliomas (HG). BNCT is a biologically targeted particle therapy that utilizes high linear energy transfer (LET) particles resulting from the reaction of low-energy thermal neutrons with boron-10 (^10^B) atoms. The cell-killing effect is limited to cells containing boron-10 since the generated particles have a path length of approximately 4–9 μm inside tissue. In other words, BNCT is very suitable for the treatment of HG, because it can prevent damage to normal brain tissues when the boron-10 compounds can accurately target glioma cells [1,2,3,4,5,6,7,8].

Previously, boronophenylalanine (BPA) and sodium borocaptate (BSH), have been clinically applied [9,10]. BPA is the only compound approved by the Japanese pharmaceutical affairs office for accelerator-based BNCT [7,11]. BPA reaches the tumor via LAT1 [12]; however, the clinical therapeutic effect of BNCT using BPA may be limited in some cases due to the non-uniform distribution of BPA in glioma cells [13,14,15,16]. BSH can only pass through the destruction of the blood–brain barrier (BBB) [17]; however, BSH cannot accumulate in the cells which have intact BBB around the brain tumor. Therefore, to improve the efficacy of BNCT, the development of novel biologically targeted boron-10 compounds that are higher than BPA and BSH needs to be developed.

We focused on the 18 kDa translocator protein (TSPO) expression in HG and its surrounding environment [18]. TSPO is a five-transmembrane domain protein of 169 amino acid residues located in the outer mitochondrial membrane, formerly known as the peripheral benzodiazepine receptor (PBR) [19,20,21]. The TSPO expression in gliomas has already been evaluated using positron emission tomography and single photon emission computed tomography (SPECT) [22,23,24,25]. The higher the TSPO expression, the higher WHO grading of gliomas, and thus there is a negative correlation between TSPO expression and survival and a positive correlation with tumor proliferative potential [26]. Moreover, PET with TSPO even has the potential to predict the prognosis of patients with recurrent gliomas [27]. We predicted that TSPO, which is highly expressed in HG and its surrounding environment, could be a new biological target for BNCT against HG, and thus developed a novel boron-10 compound. In this study, a dodecaborated compound targeting TSPO, named DPA-BSTPG, has been developed and evaluated the potential of BNCT targeting TSPO against HG.

## 2. Materials and Methods

### 2.1. Boron Compounds

**Figure A1:**
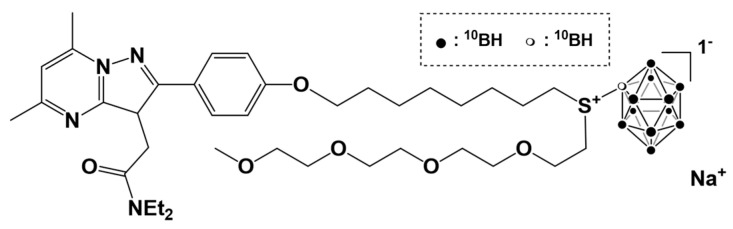
The chemical structure of DPA-BSTPG.

DPA-BSTPG was synthesized by the method already reported [28]. The chemical structure of DPA-BSTPG is shown in Figure A1. BPA (L-isomer) was provided by STELLA PHARMA CORPORATION (Osaka, Japan) and converted into fructose complex [29]. BSH was purchased from Katchem (Prague, Czech Republic) and dissolved in sterile saline. All boron compounds used isotopically enriched in ^10^B.

### 2.2. Cell Culture

F98 rat glioma cells were provided by Dr. Rolf Barth (The Ohio State University, Columbus, OH, USA). C6 rat glioma cells were purchased from the Japanese Collection of Research Bioresources (JCRB) Cell Bank, National Institute of Biomedical Innovation (Osaka, Japan). 9L rat glioma cells were purchased from the American Type Culture Collection (ATCC) (Manassas, VA, USA). They were cultured at 37 ℃ in a 5% CO_2_ atmosphere on Dulbecco’s Modified Eagle’s Medium (DMEM) supplemented with 10% fetal bovine serum and penicillin, streptomycin, and amphotericin B. These were purchased from Gibco Invitrogen (Grand Island, NY, USA).

### 2.3. The F98 Rat Glioma Bearing Brain Tumor Model and the Implantation of the Alzet Osmotic Pump

All animal experiments were performed in accordance with the guide for the care and use of laboratory animals approved by the Animal Use Review Board and Ethical Committee of Osaka Medical and Pharmaceutical University (License No. 30036) and the Institute for Integrated Radiation and Nuclear Science, Kyoto University (KURNS; Kumatori, Osaka, Japan) (License No. 2018-9). Male Fischer rats that were 10 weeks old weighed between 200 and 250 g (Japan SLC, Shizuoka, Japan) were anesthetized by intraperitoneal injection of the mixed anesthetics which were previously used in our group [30,31,32,33,34] and fixed with a stereotactic frame (Model 900, David Kopf Instruments, Tujunga, CA, USA); 10^3^ F98 rat glioma cells diluted in a 10 μL solution of DMEM containing 1.4% agarose (Wako Pure Chemical Industries, Osaka, Japan) for therapeutic experiments or 10^5^ F98 rat glioma cells for biodistribution experiments and real-time polymerase chain reaction (PCR) were injected at a rate of 20 μL/min by an automated infusion pump. The Alzet osmotic pump (model #2001D, Durect, Cupertino, CA, USA) for convection-enhanced delivery (CED) were implanted in our procedure [30,31,32,33]. The F98 rat glioma bearing brain tumor model is referred to here as the F98 rat glioma model.

### 2.4. Cellular Uptake of Boron in Rat Glioma Cell Lines

On a 100 mm dish, 4 × 10^5^ F98, C6, or 9L rat glioma cells were seeded and grown (Becton, Dickinson, and Company, Franklin Lakes, NJ, USA). Subsequently, they were incubated for 24 h with each medium containing 2.5 μg boron (B)/mL of DPA-BSTPG, BPA or BSH. Each dish was washed twice with phosphate-buffered saline (PBS) and detached using trypsin-ethylenediamine tetraacetic acid solution. Cell numbers for each experiment were counted after two centrifugations (200× *g*, 5 min). They were digested overnight with 1N nitric acid solution (Wako Pure Chemical Industries, Osaka, Japan). The boron-10 concentrations were measured by using inductively coupled plasma atomic emission spectroscopy (ICP-AES; iCAP6300 emission spectrometer, Hitachi, Tokyo, Japan). Results were normalized as μg B/10^9^ cells.

### 2.5. Estimating the Compound Biological Effectiveness on an In Vitro Neutron Irradiation Experiment

F98 rat glioma cells were incubated on the medium containing 2.5 μg B/mL of DPA-BSTPG, BPA, or BSH for 24 h. After this incubation, they were irradiated using the nuclear reactor at KURNS at a reactor power of 1 MW with the Heavy Water Irradiation Facility for 10, 20, or 30 minutes, and then incubated in a 60-mm dish (Becton, Dickinson. and Company, Franklin Lakes, CA, USA) for 7 days. They were fixed with 10% formalin and stained with trypan blue. The survival fraction (SF) was calculated by dividing the number of colonies containing more than 50 cells by the number of colonies in the control group. These results were compared with the linear quadratic (LQ) model estimated with the SF obtained by X-ray irradiation using a photon radiation device (M-150WE; SOFTEX, Tokyo, Japan). The relative biological effectiveness (RBE) of the neutron beam (RBE-beam) and the compound biological effectiveness (CBE) were calculated from the absorbed dose at SF = 0.1. CBE is a specific relative biological effectiveness factor for the radiation component due to boron neutron capture reaction. In other words, CBE is derived from two factors: the RBE of α-particles and ^7^Li ions and the micro-distribution of ^10^B in a particular tissue. Because of the short range of these particles in tissues, the biological effect depends critically on both the gross and microscopic distributions of ^10^B in tissues [35].

### 2.6. Evaluating the TSPO Expression in F98 Rat Glioma Model

Each tumor and tumor contralateral brain tissue were removed at 12 days post-implantation of 10^5^ F98 rat glioma cells. The RNA was extracted using miRNeasy Mini Kit (Qiagen, Hilden, Germany), and cDNA library preparation was performed using a SuperScript VLIO cDNA Synthesis kit (Thermo Fisher Scientific, Cleveland, USA) [36]. Real-time PCR was performed by using the system of StepOnePlus™ (applied Biosystems, Carlsbad, USA) according to the manufacturer’s instructions. Predesigned TaqMan fluorogenic probes and primer sets for peripheral benzodiazepine receptor (PBR) (Rn00560892_m1) and β-actin (Rn00667869_m1) (applied Biosystems, Foster City, CA, USA). The cycle threshold (CT) values were analyzed using the 2^-ΔΔCT^ method. In each individual, the expression ratio of TSPO and β-actin in normal brain and brain tumor was measured using the 2^-ΔΔCT^ method. Subsequently, the values were compared between the normal brain and the brain tumor.

### 2.7. Biodistribution of Boron Compounds in F98 Rat Glioma Models

Approximately 12 days post-implantation of F98 rat glioma cells, each boron compound was administered at the following body-weight (b.w.) doses: 250 mg/kg of BPA (12 mg B/kg b.w.) in BPA group and 84.1 mg/kg of DPA-BSTPG (12 mg B/kg b.w.) in DPA-BSTPG group by intravenous administration (i.v.) or 4.2 mg/kg of DPA-BSTPG (0.6 mg B/kg b.w.) in DPA-BSTPG group by CED administration, respectively. Each rat was euthanized and the tumor, brain, blood, heart, lung, liver, kidney, spleen, skin, and muscle were removed. Each organ was weighed and digested with 1N nitric acid solution. After each organ dissolved, the boron concentrations were measured by using ICP-AES. Results were normalized as μg B/g. The tissue boron concentrations in the combined BPA and DPA-BSTPG group was measured at the time when the boron concentration of each single compound was determined to be optimal for neutron irradiation, referring to the accumulation of boron from the administration of each boron compound alone. In other words, the boron concentrations in the combined BPA and DPA-BSTPG group were measured under conditions of 2.5 h after i.v. of BPA and 2.5 h after CED administration of DPA-BSPTG based on the results of dosing experiments for each boron compound.

### 2.8. Survival Analysis of F98 Rat Glioma Models on an In Vivo Neutron Irradiation Experiment

After 14 days post-implantation of F98 rat glioma cells, 28 rats were randomly divided into the following 5 groups; untreated group (Untreated), neutron irradiated group (Neutron only), neutron irradiation following BPA i.v. group (BPA-BNCT), neutron irradiation following DPA-BSTPG CED administration group (DPA-BSTPG-BNCT), neutron irradiation following combination of DPA-BSTPG CED administration and BPA i.v. group (Combination BNCT). The irradiation was performed 2.5 h after the termination of administration. Excluding their heads, each rat body was attached on a plate lined with ^6^LiF ceramic tiles to shield and reduce neutron irradiation, and then neutron irradiation was performed. They were irradiated at a reactor power of 1 MW for 1 h at KURNS. They were observed until death or euthanasia. The therapeutic effects were evaluated by Kaplan–Meier survival curves and the percentage of increased lifespan (%ILS) [37]. The percent increased life span (%ILS) was defined as the relative value to the median survival times (MST) of the Untreated group; %ILS was calculated by the equation (MST of each BNCT group—MST of the Untreated group) × 100/(MST of the Untreated group). The absorbed doses and estimated photon equivalent doses were calculated by the RBE-beam and CBE obtained from the in vitro neutron irradiation experiment based on our previous studies [31,32,33,34]. In this study, the prescribed doses were fixed irradiations at 1 MW 1 h. Based on this fixed neutron irradiation dose, the prescribed dose was calculated according to the boron concentration of each tissue. The prescribed dose of BPA to normal brain tissue was calculated based on the existing CBE values for normal brain tissue [1], but the CBE of DPA-BSTPG to normal brain tissue was unknown and could not be experimentally estimated in this study.

### 2.9. Doses Calculation

Each irradiated cell was irradiated using the nuclear reactor at KURNS at a reactor power of 1 MW for 10, 20, or 30 minutes. Each of the irradiated animals was irradiated at a reactor power of 1 MW for 1 h at KURNS. The total absorbed dose was calculated by summing the four main dose components, D_B_ + D_N_ + D_H_ + D_γ_, which correspond to the ^10^B (n,α) ^7^Li, ^14^N (n,p) ^14^C, ^1^H (n,n) ^1^H, and ^1^H (n, γ) ^2^H neutron reactions, respectively. D_B_ is the absorbed dose of boron derived from the equation of 7.43 × 10^−14^ (Gy cm^2^/ μg ^10^B/g) × boron concentration (μg ^10^B/g) × thermal neutron fluence (1/cm^2^). D_N_ is the absorbed dose of nitrogen derived from the equation of 6.78 × 10^−14^ (Gy cm^2^/weight %) × nitrogen concentration (weight %) × thermal neutron fluence (1/ cm^2^). D_H_ is the elastic scattering between epithermal or fast neutrons and the hydrogen nucleus, and D_γ_ is the γ-ray dose resulting from both the γ-rays emitted when a hydrogen atom captures a thermal neutron and γ-rays from the source itself. The estimated photon-equivalent doses were calculated by the equation D_B_ × compound biological effectiveness (CBE) + D_N_ × relative biological effectiveness of nitrogen (RBE_N_) + D_H_ × relative biological effectiveness of hydrogen (RBE_H_) + D_γ_. In this study, RBE-beam calculated from in vitro experiments was used for RBE_N_ and RBE_H_. Doses to the F98 rat glioma bearing brain tumor models were corrected based on our previous reports [31].

### 2.10. Statistical Analysis

Cellular uptakes of boron were compared using Student’s *t*-tests. The log-rank test was used to determine the significant difference at each BNCT group. All *p*-values less than 0.05 were considered statistically significant. Statistical analyses were performed using the JMP® Pro version 15.1.0. software program (SAS, Cary, NC, USA). The datasets analyzed during this study are available from the corresponding author on reasonable request.

## 3. Results

### 3.1. Cellular Uptake of Boron in Rat Glioma Cell Lines

**Figure 1:**
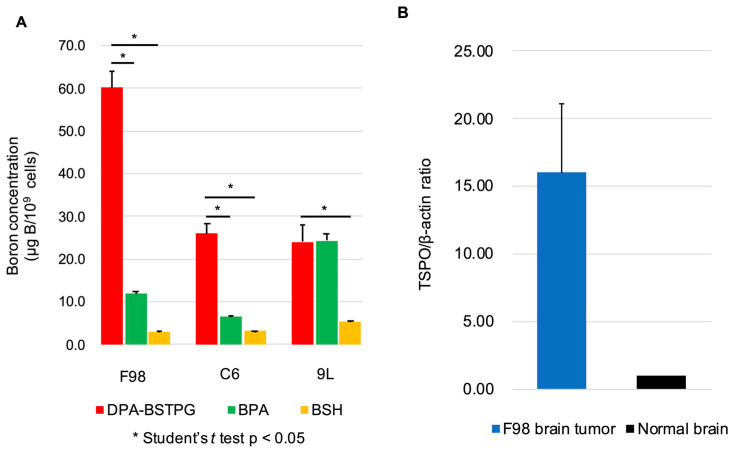
(**A**) This figure shows the cellular uptakes of boron using three boron-10 compounds. DPA-BSTPG, BPA, or BSH for F98 glioma cell lines were 60.3 ± 3.8, 11.9 ± 0.5, or 3.0 ± 0.2 μg boron B/10^9^ cells, respectively. In C6 rat glioma cells, 26.0 ± 2.4, 6.5 ± 0.2, or 3.2 ± 0.1 μg B/10^9^ cells, respectively. In 9L rat glioma cells, 24.0 ± 4.0, 24.3 ± 1.7, or 5.3 ± 0.4 μg B/10^9^ cells, respectively. In F98 and C6 rat glioma, DPA-BSTPG was the highest boron uptake, with statistically significant uptake compared to BPA (F98; *p* < 0.0001, C6; *p* = 0.0003, respectively) or BSH (F98; *p* < 0.0001, C6; *p* = 0.0002, respectively), while BPA was the highest boron uptake in 9L rat glioma (vs. DPA-BSTPG; *p* = 0.93) and DPA-BSTPG had statistically significant uptake compared to BSH (*p* = 0.0027). (**B**) The TSPO expression of each F98 brain tumor was evaluated. The ratio of TSPO to β-actin between F98 rat brain tumor to normal brain in an F98 rat glioma model was calculated using the 2^-ΔΔ^CT method with real-time PCR in F98 rat glioma models. The ratios were 13.95, 23.07, and 11.09, respectively, indicating that the TSPO expression was 16-hold higher on average in the F98 brain tumor than in the normal brain.

The cellular uptakes of boron concentrations using DPA-BSTPG, BPA, or BSH were 60.3 ± 3.8, 11.9 ± 0.5, or 3.0 ± 0.2 μg B/10^9^ cells in F98, 26.0 ± 2.4, 6.5 ± 0.2, or 3.2 ± 0.1 μg B/10^9^ cells in C6, and 24.0 ± 4.0, 24.3 ± 1.7, or 5.3 ± 0.4 μg B/10^9^ cells in 9L. The cellular uptakes of boron concentration using DPA-BSTPG showed statistically significant differences compared to BPA or BSH by Student’s *t*-tests in F98 and C6. These results were shown in Figure 1A. Each of these units “μg B/10^9^ cells”, based on weight, can be approximated to “ppm” units if they are converted.

### 3.2. The TSPO Expressions in the F98 Rat Glioma Model

The TSPO expression evaluation in three F98 rat glioma models were 13.95, 23.07, and 11.09, respectively, by using the 2^-ΔΔCT^ method. The TSPO expression was 16-fold higher in the tumor than the normal brain tissues. These data are shown in Figure 1B.

### 3.3. Estimating the Compound Biological Effectiveness on an In Vitro Neutron Irradiation Experiment

**Figure 2:**
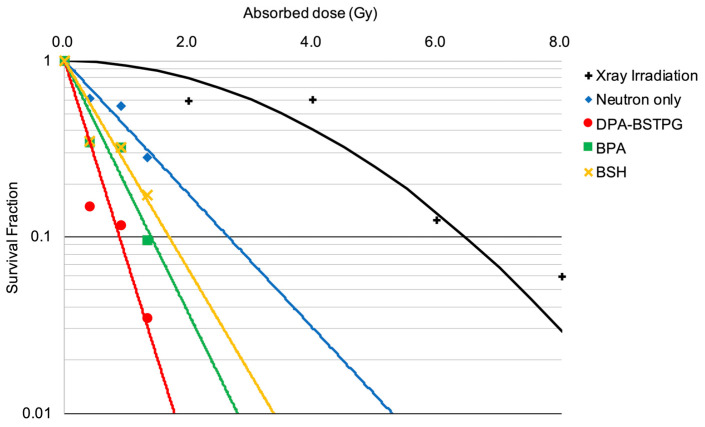
The absorbed doses required to obtain at survival fraction (SF) = 0.1 were 6.45 Gy in the LQ model, 2.63 Gy in the mixed neutron beam alone, 1.39 Gy in the BPA group, and 0.88 Gy in the DPA-BSTPG group, respectively. In the Neutron only curve at SF = 0.1, the dose exerted on γ-rays component mixed with neutron irradiation was excluded from 6.45 Gy, and the relative biological effectiveness (RBE) to the mixed neutron beam (RBE-beam) was calculated since there was no dose contributed by boron-10. As a result, the RBE-beam was estimated as 3.00. The CBE for F98 rat glioma cell of BPA, BSH, and DPA-BSTPG were 3.80, 2.41 and 8.43 respectively.

In the neutron irradiation experiment, the SF obtained by each boron-10 compound decreased as the absorbed dose increased. The SF obtained using the mixed neutron beam alone was compared to that of the LQ model. The RBE-beam was calculated by excluding the effect of the absorbed dose from the boron and γ-rays. Consequently, the absorbed doses required to obtain an equivalent biological effect at SF = 0.1 for the LQ model, the mixed neutron beam alone, the DPA-BSTPG group, the BPA group, and the BSH group were 6.45 Gy, 1.39 Gy, 0.88 Gy, 2.63 Gy, and 1.68 Gy, respectively. In addition, the RBE-beam was estimated to be 3.0. For the F98 rat glioma cell, the estimated CBE for DPA-BSTPG, BPA, or BSH was 8.43, 3.80, or 2.41, respectively. All SFs are shown in Figure 2.

### 3.4. Biodistribution of Boron Compounds in F98 Rat Glioma Models

The boron concentrations in the tumor at 2.5, 6, and 24 h after DPA-BSTPG CED administration was evaluated since the boron concentration in the tumor was insufficient by i.v.. In fact, the boron concentration in each organ after DPA-BSTPG i.v. was 2.3 ± 0.3 in the tumor at 2.5 h after i.v. The boron concentrations in the tumor, brain, and blood at 2.5 h after CED administration of DPA-BSTPG were 45.0 ± 18.8, 0.8 ± 0.8, and 0.4 ± 0.2, respectively. The boron concentration using DPA-BSTPG in each organ gradually decreased over time. The biodistribution experiment in combination of BPA and DPA-BSTPG administration was conducted 2.5 h after completion of the administration, when the two boron-10 compounds had the highest boron concentrations in the tumor. The boron concentrations in the tumor, brain, and blood at 2.5 h after the completion of the administration were 61.8 ± 20.4, 5.1 ± 0.7, and 7.3 ± 0.5, respectively. In addition, the boron concentration in systemic organs by CED administration of DPA-BSTPG was extremely low level as shown in Table A1, and no apparent adverse events occurred in the F98 rat glioma models after the observation period. Summary of the boron concentration after administration of each boron compound is shown in Table 1. The boron concentration using BPA i.v. in each organ was based on our previous report [34].

### 3.5. Survival Analysis of the F98 Rat Glioma Model on an In Vivo Neutron Irradiation Experiment

**Figure 3:**
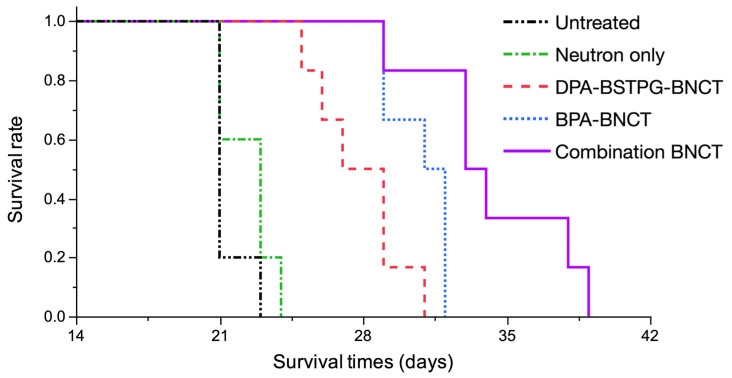
The survival times in days after implantation of 10^3^ F98 rat glioma cells have been plotted. Five groups were prepared: Untreated, Neutron only, DPA-BSTPG-BNCT, BPA-BNCT, and Combination BNCT. There were statistically significant differences between three BNCT groups and the Untreated group (vs. DPA-BSTPG-BNCT, *p* = 0.0008; vs. BPA-BNCT, *p* = 0.0008; vs. Combination BNCT, *p* = 0.0008, respectively, log-rank test), with all BNCT groups showing prolonged survival times. There was a statistically significant difference observed between BPA-BNCT and DPA-BSTPG-BNCT (*p* = 0.0187, log-rank test). On the other hand, a statistically significant prolongation of survival times was observed in Combination BNCT compared to BPA-BNCT (*p* = 0.0121, log-rank test). In this study, Combination BNCT obtained the longest survival among all groups.

The treatment efficacy was evaluated using Kaplan–Meier survival curves. Each median survival time (MST) was as follows: Untreated; 21.0 days [95% confidence interval (CI); 21–23 days], Neutron only; 23.0 days [95% CI; 21–24 days], DPA-BSTPG-BNCT; 28.0 days [95% CI; 25–31 days], BPA-BNCT; 31.5 days [95% CI; 29–32 days], and Combination BNCT; 33.5 days [95% CI; 29–39 days] were observed, respectively. Statistically significant differences were observed between Untreated and BNCT groups (*p* = 0.0008, log-rank test). Each %ILS value was as follows: Neutron only; 9.5%, DPA-BSTPG-BNCT; 33.3%, BPA-BNCT; 50.0%, and Combination BNCT; 59.5%, respectively. The absorbed dose and photon equivalent dose of DPA-BSTPG were based on 26.8% boron contribution from CED administration [32]. The estimated photon-equivalent doses of DPA-BSTPG-BNCT or BPA-BNCT were 11.8 or 10.0 Gy-Eq, respectively. All results are shown in Figure 3 and Table 2.

## 4. Discussion

In BNCT, a high LET radiation therapy with selectivity at the cellular level, it is important to consider the possibility that some cancer cells may not be targeted due to heterogeneous distribution of boron-10 compounds. The combination of multiple boron compounds is thought to be a means of resolving this issue, and this basic study demonstrates the utility of the simultaneous use of TSPO-targeted boron compound (DPA-BSTPG) and BPA, which target tumor tissue by different mechanisms.

In this study, the cellular level uptake of DPA-BSTPG was found to be superior to those of BPA and BSH in F98 and C6 rat glioma cells, revealing the potential for drug delivery by targeting TSPO. BPA was found to accumulate most efficiently for 9L rat glioma cells, while the accumulation of DPA-BSTPG was not higher than that of BPA for 9L rat glioma cells, unlike the other two cell lines. The 9L rat glioma cells may benefit from BPA-BNCT, but may benefit less from TSPO-targeted BNCT. Conversely, F98 and C6 rat glioma cells may be more likely to benefit not only from BPA-BNCT, but also from TSPO-targeted BNCT. In other words, since HG in clinical settings is a heterogenous tumor that combines the individual characteristics of, for example, F98, C6, and 9L rat glioma cells, multi-targeted neutron capture therapy that can utilize not only LAT1 which is targeted by BPA but also other targets may be effective.

From another perspective, not only evaluating boron uptake at the cellular level, but also estimating CBE value, which is uniquely determined for each combination of irradiated tissue and boron compound in the neutron capture reaction, is essential to evaluate the boron-10 compound. In this study, the CBE values for F98 rat glioma cells were estimated to be 8.43 for DPA-BSTPG, 3.80 for BPA, and 2.41 for BSH. The RBE-beam value estimated in this study was 3.00. The CBE values of BPA and BSH for HG and the RBE-beam value were comparable to those used in clinical trials [1]. However, the CBE value is an evaluation used for intravenous administration, and it is debatable whether it can be used to the same extent for CED administration. In fact, the contribution of boron concentration in CED administration is estimated to be approximately 26% [32], and further clarification is needed.

The TSPO expression was found to be localized to the tumor in the F98 rat brain tumor model and was predicted to be a valuable biological target for BNCT. Generally, TSPO is known to be highly expressed in glioblastoma and surrounding immune cells, such as macrophages and microglia [18,38], and is visualized by targeting TSPO in PET imaging. The tracer targeting TSPO suggests an association between the TSPO expression level and isocitrate dehydrogenase (IDH) mutation [39,40], which is associated with glioma prognosis, furthermore, the SPECT targeting TSPO has the potential to predict recurrence of HG before the discovery of a recurrence as an enhanced lesion with standard contrasted magnetic resonance imaging [22]. In the most recent report, PET with TSPO can even predict the prognosis of glioma patients [27]. Therefore, in the future, TSPO would be exploited as a therapeutic target for HG likely to recur from residual tumor cells. In our research group, an experimental F98 rat glioma model that has been shown to successfully replicate the microenvironment of a human HG was used for evaluation [41,42]. The TSPO, which has been shown to be expressed in the C6 rat glioma bearing brain tumor model [43,44] and 9L rat glioma bearing brain tumor model [45,46], was also confirmed in the F98 rat glioma model. As a result, an F98 rat glioma model was shown to be suitable for experiments targeting TSPO. Since TSPO expression was confirmed in the F98 rat glioma model, DPA-BSTPG, which was developed to target TSPO, could be administered using CED to deliver boron to the tumor. Compared to BPA, the CED administration of DPA-BSTPG achieved boron concentrations about twice as high as when BPA was administered, and the boron concentration was sufficient for BNCT experiments [30,31,32,33,37]. In other words, the feasibility of the TSPO-targeted BNCT experiment using the F98 rat glioma model was proven.

In the BNCT experiment, the %ILS of DPA-BSTPG-BNCT was lower in BPA-BNCT; however, the estimated photon-equivalent dose of DPA-BSTPG-BNCT was higher than that of BPA-BNCT shown in Table 2. These results may be due to the complicated compound distribution. First, due to the unique boron delivery method which CED administration has, there were glioma cells achieving lower boron concentrations than anticipated [33]. In other words, it is possible that not all of the tissue boron distributed in the CED administration was used in the boron neutron capture reaction. TSPO is expressed not only in glioblastoma but also in immune cells, such as macrophages and microglia, in the glioblastoma periphery. Therefore, DPA-BSTPG was probably distributed to these cells as well. Furthermore, it is not even clear whether these peritumoral cells suppress or promote tumor immunity [38,47,48], indicating that the destruction of these cells would cause complicated reactions. However, with these preliminary experiments in this study, it was not possible to understand if boron distribution in the microenvironment might have a role in determining the therapeutic effect; thus, we would like to elucidate this mechanism in the future. Although macroscopic evaluations of compound distribution administering CED have been evaluated so far in our group [30,31,32,33,49], detailed evaluations of the micro-distribution of boron compounds in the brain tumor and the peri-tumor region are essential to evaluate boron neutron reaction accurately, and this would be a new research challenge.

On the other hand, BNCT using the combination of CED administration of DPA-BSTPG and intravenous BPA administration was more effective than BPA-BNCT because the different biological targets were able to deliver boron neutron capture responses to a wider range of glioma cells. It is already known that BPA is non-uniformly distributed in gliomas when BPA is administered [13,14,15,16], and furthermore, glioblastoma has proven inter-individual heterogeneity of TSPO and LAT1 expression in neoplastic and parenchymal cells [40]. In this study, the combination treatment would have distributed DPA-BSTPG to compensate for the heterogeneous accumulation of BPA to glioma cells, resulting in the reduction in the number of the glioma cells with insufficient BPA accumulation, so-called BPA-refractory glioma cells, which would have enhanced the BNCT effect. In other words, the development of the boron-10 compound that can be used in combination with BPA and the establishment of the method, which was CED, for the administration could be found to enable multi-targeted neutron capture therapy and also to improve the efficacy of BNCT using BPA. Within the scope of this observation, no obvious changes in F98 rat glioma bearing brain tumor models were observed in the short period of time after DPA-BSTPG administration. In the future, it seems urgent to clarify the distribution of boron in the microenvironment around HG by CED administration, which can be used for clinical trials, and to develop a detailed experimental plan for toxicity testing, in order to put DPA-BSTPG into practical use.

## 5. Conclusions

The brain tumor in an F98 rat glioma model expressed TSPO. DPA-BSTPG, a boron-10 compound targeting TSPO, provided an effective BNCT against the F98 rat glioma model. The combination of BPA and DPA-BSTPG would add a novel biological target and destroy BPA-refractory glioma cells. This study showed that DPA-BSTPG in combination with BPA may provide the multi-targeted neutron capture therapy against HG. The multi-targeted neutron capture therapy may play an important role in BNCT for HG.

## Figures and Tables

**Table 1:** Summary of the boron concentrations after administration of each boron compound on the F98 rat glioma model.

Boron Compound ^a^	Time ^b^	*n* ^c^	Boron Concentrations ± SD (μg B/g) ^d^			Ratio	
			Tumor	Brain	Blood	T/Br ^e^	T/Bl ^f^
DPA-BSTPG	2.5	6	45.0 ± 18.8	0.8 ± 0.8	0.4 ± 0.2	55.0	115.1
	12	4	32.5 ± 13.1	0.8 ± 0.8	0.3 ± 0.1	40.7	106.8
	24	4	23.7 ± 13.5	1.0 ± 1.4	0.4 ± 0.1	23.5	61.5
BPA	2.5	4	20.6 ± 2.2	5.5 ± 0.6	7.7 ± 0.5	3.8	2.7
	12	4	9.1 ± 3.3	2.5 ± 0.6	2.9 ± 0.4	3.7	3.2
	24	4	8.2 ± 0.8	2.3 ± 0.3	2.9 ± 0.4	3.6	2.8
Comnination ^g^	2.5	4	61.8 ± 20.4	5.1 ± 0.7	7.3 ± 0.5	12.1	8.5

^a^ Doses: 250 mg/kg of BPA (12 mg B/kg b.w.) in BPA group by intravenous administration and 4.2 mg/kg of DPA-BSTPG (0.6 mg B/kg b.w.) in DPA-BSTPG group by CED administration for 8 μL/h, respectively. ^b^ Time indicates the time (h) from the termination of CED administration. ^c^ n indicates number of Fischer rats. ^d^ Boron concentration ± SD is measured by ICP-AES for the accumulated boron concentration of each organ listed in the table, and is expressed as the mean boron values (μg B / gram: weight of organ) ± standard deviation. ^e^ T/Br indicates the tumor to brain ratio. ^f^ T/Bl indicates the tumor to blood ratio. ^g^ Combination was a group that received an intravenous administration of BPA and CED administration of DPA-BSTPG. The experiments were performed by euthanizing the animals at 2.5 h after completion of the two boron-10 compound administrations.

**Table 2:** Summary of the absorbed dose, the estimated photon-equivalent dose, the estimated compound biological effectiveness (CBE), and the percentage of increased lifespan (%ILS) for the brain and the tumor in the F98 rat glioma model on the neutron irradiation experiment.

Group	Absorbed Dose ^a^ (Gy)		CBE ^b^	Photon-Equivalent Dose ^c^ (Gy-Eq)		%ILS ^d^
	Brain	Tumor		Brain	Tumor	
Untreated	0.0	0.0	-	0.0	0.0	-
Neutron only	0.9	0.9	-	1.5	1.5	9.5
DPA-BSTPG-BNCT	1.0	2.2	8.43	-	11.8	33.3
BPA-BNCT	1.5	3.2	3.80	2.3	10.0	50.0
Combination BNCT	-	-	-	-	-	59.5

^a^ The absorbed dose depends on the ^10^B (n,α) ^7^Li, ^14^N (n,p) ^14^C, and ^1^H (n,n) ^1^H reactions produced by the thermal, epithermal, and fast neutron fluxes, and γ-rays in the irradiated neutrons. It is calculated by using the following equation: Absorbed dose (Gy) = D_B_ + D_N_ + D_H_ + D_γ_. ^b^ The estimated compound biological effectiveness (CBE) was calculated by the in vitro neutron irradiation experiment on F98 rat glioma cells. The estimated CBE for the F98 rat glioma cell of DPA-BSTPG was 8.43 and that of BPA was 3.80. ^c^ The estimated photon-equivalent dose is calculated by using the following equation: D_B_ × compound biological effectiveness (CBE) + D_N_ × relative biological effect of nitrogen (RBE_N_) + D_H_ × relative biological effect of hydrogen (RBE_H_) + D_γ_. The RBE-beam value of 3.00 calculated by the in vitro neutron irradiation experiment on F98 rat glioma cells was used as RBE_N_ and RBE_H_. Since CBE in normal brain tissue cannot be measured, a value of 1.35 was used for BPA [1]. The CBE of DPA-BSTPG to normal brain tissue was unknown and could not be experimentally estimated in this study. ^d^ The percent increased life span (%ILS) was defined as the relative value to the median survival times (MST) of the Untreated group. %ILS was calculated using the following equation (MST of each BNCT group—MST of Untreated group) × 100/(MST of Untreated group).

## Data Availability

The datasets analyzed during this study are available from the corresponding author on reasonable request. The JMP® Pro version 15.1.0. software (SAS, Cary, NC, USA) was used for statistical analysis.

## Appendix A

**Table A1:** Summary of the boron concentrations of systemic organs after administration of DPA-BSTPG on the F98 rat glioma model.

Boron Compound ^a^	Time ^b^	*n* ^c^	Boron Concentrations ± SD (μg B/g) ^d^								
			Brain	Blood	Heart	Lung	Liver	Kidney	Spleen	Skin	Muscle
DPA-BSTPG	2.5	6	0.8 ± 0.8	0.4 ± 0.2	0.1 ± 0.1	0.2 ± 0.1	0.3 ± 0.1	0.1 ± 0.0	0.2 ± 0.0	0.0 ± 0.0	0.0 ± 0.0
	12	4	0.8 ± 0.8	0.3 ± 0.1	0.1 ± 0.0	0.1 ± 0.0	0.4 ± 0.2	0.1 ± 0.0	0.1 ± 0.0	0.0 ± 0.0	0.0 ± 0.0
	24	4	1.0 ± 1.4	0.4 ± 0.1	0.2 ± 0.0	0.2 ± 0.1	0.3 ± 0.0	0.2 ± 0.0	0.2 ± 0.0	0.1 ± 0.0	0.1 ± 0.0

^a^ Dose: DPA-BSTPG (0.6 mg B/kg b.w.) in DPA-BSTPG group by CED administration for 8 μL/h. ^b^ Time indicates the time (h) from the termination of CED administration. ^c^ n indicates number of Fischer rats. ^d^ Boron concentration ± SD is measured by ICP-AES for the accumulated boron concentration of each organ listed in the table, and is expressed as the mean boron values (μg B/gram: weight of organ) ± standard deviation.
